# Answers to naysayers regarding microbial extracellular vesicles

**DOI:** 10.1042/BST20180252

**Published:** 2019-07-18

**Authors:** Carolina Coelho, Arturo Casadevall

**Affiliations:** 1 Medical Research Council Centre for Medical Mycology, Department of Biosciences, College of Life and Environmental Sciences, University of Exeter, Exeter, U.K.; 2 Institute of Medical Sciences, University of Aberdeen, Aberdeen, U.K.; 3 Department of Molecular Microbiology and Immunology, Johns Hopkins Bloomberg School of Public Health, Baltimore, MD, U.S.A

## Abstract

It is now over 30 years since the discovery of extracellular vesicles (EVs) in Gram-negative bacteria. However, for cell-walled microbes such as fungi, mycobacteria and Gram-positive bacteria it was thought that EV release would be impossible, since such structures were not believed to cross the thick cell wall. This notion was disproven 10 years ago with the discovery of EVs in fungi, mycobacteria, and gram-positive bacteria. Today, EVs have been described in practically every species tested, ranging from Fungi through Bacteria and Archaea, suggesting that EVs are a feature of every living cell. However, there continues to be skepticism in some quarters regarding EV release and their biological significance. In this review, we list doubts that have been verbalized to us and provide answers to counter them. In our opinion, there is no doubt as to existence and physiological function of EVs and we take this opportunity to highlight the most pressing topics in our understanding of the biological processes underlying these structures.

## EV discovery

**Figure 1.**
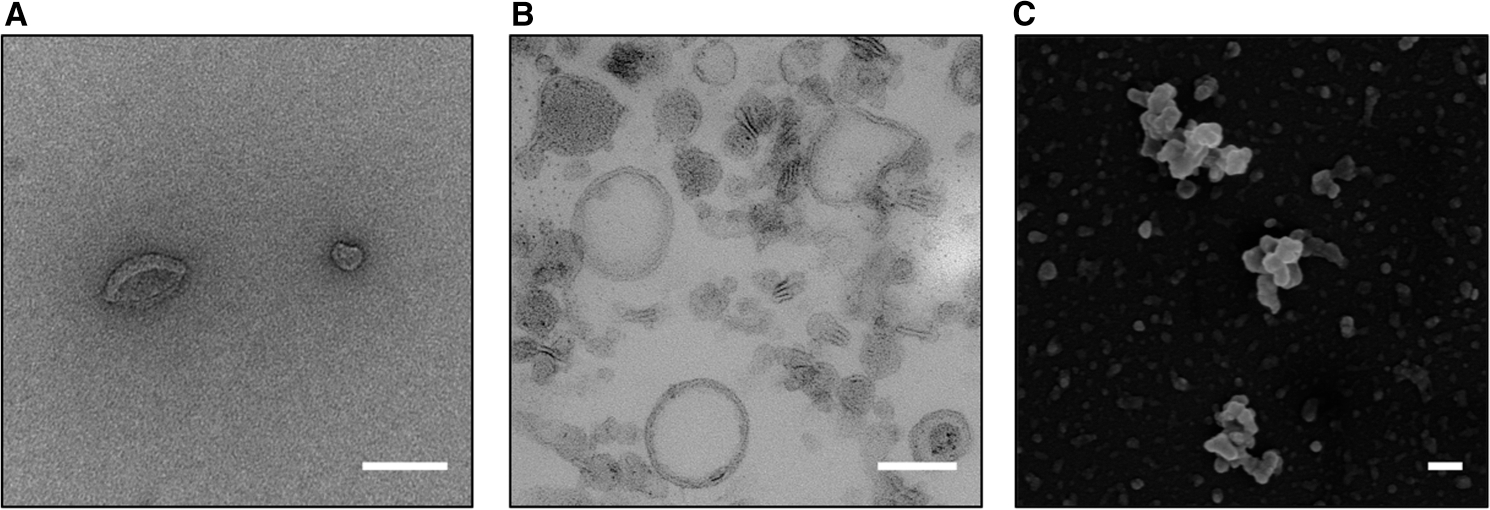
EVs from Gram + bacteria and fungi. (**A**) Negative Staining electron micrograph of *L. monocytogenes* EVs after Optiprep gradient separation (as prepared in [30]). (**B**) Transmission electron micrograph of EVs from *C. neoformans* (as prepared in [58]). (**C**) Scanning electron micrograph of EVs from *C. neoformans* (as prepared in [58]). Scale bars 100 nm. Images courtesy of Carolina Coelho and Raghav Vij (**A**), and Julie M. Wolf (**B** and **C**).

Extracellular vesicles (EVs) are broadly classified as: ‘particles naturally released from the cell that are delimited by a lipid bilayer and cannot replicate’ [1]. It is now over 30 years since the discovery of EVs in Gram-negative bacteria [2]. Due to the lack of an outer membrane layer and the presence of thick cell wall it was thought that other microbes, such as Gram-positive bacteria, as well as fungi, could not release EVs. It took almost 20 years for EVs to be discovered in mycobacteria [3], fungi [4], Gram-positive bacteria [5], and Archaea [6] (Figure 1). Microbial EVs are defined as ‘spherical, membranous vesicles from microbial cell surfaces’ [7] and ‘ranging in size from 20 to 500 nm in diameter’ [8]. EVs from Gram-negative bacteria are referred to as outer membrane vesicles (OMV), and for the purpose of this review, we will consider OMV as a sub-class of microbial EVs. EV release in all domains of life suggests that EVs are a primordial feature of living cells.

The discovery of EVs was a breakthrough in the field of secretion as it provided a new mechanism for the release of components into the extracellular milieu. While the majority of the molecular mechanisms underlying EV release and cargo loading into the EVs are still unknown [7–10], this is still a recent field and will certainly be elucidated in the coming years.

Microbial EVs from pathogens carry a myriad of toxins and virulence factors. EVs have been associated with pathogenicity from delivering toxins to the host as well as antibiotic resistance, partially by contributing to biofilm formation [11,12]. On the other hand, EVs have been harnessed for vaccines: EVs are protective as vaccines in mouse models for several human pathogens [13,14]. The potential of EVs as vaccines is sufficient to warrant detailed studies of EVs. (There is some confusion with the clinically approved OMV vaccines, which are extracts from the outer membrane of *Neisseria meningitidis*, further processed into a vesicular/liposomal form [15,16]. These are not EVs since they do not derive from secretion from bacterial cell.)

Despite tremendous progress in the EV field, there continues to be some skepticism as to the existence of EVs and their physiological reality. For decades bacterial EV from gram-negative bacteria were considered artifacts of bacterial growth [17]. Although great progress has been made in recent years in the acceptance of these structures as bona fide products of microbial physiology, we continue to hear apprehensions as to their physiological relevance, with some scientists arguing that EVs are experimental artifacts. The concerns can be briefly summarized as (1) bacteria do not have organelles so they do not possess the machinery to release EVs; (2) EVs are artifacts of lipid self-aggregation; (3) there are no EV-null strains, which argues against a regulated process; (4) EVs cannot cross rigid cell walls; and (5) EVs serve no function. Some of these arguments apply to all microbes, while some apply to bacteria or cell-walled organisms. To complicate the matter further, the EV field was hindered by the relatively low yields obtained as well as the lack of tools capable of analyzing or purifying these small biological particles. The EV field has strived to tackle these problems and high standards of quality. The International Society for Extracellular Vesicles has published and updated guidelines for EV research [1], and created the EV-track database for protocol publication (http://evtrack.org/index.php, [18]). In this review, we address the issues raised by skeptics while simultaneously highlighting areas requiring better experimental evidence.

## Discussion of criticisms of EVs

### Bacteria do not have organelles so they do not possess the machinery to release EVs

Simply put, the argument of skeptics is that because bacteria do not possess organelles (defined as lipid-bound compartments within the cell), bacteria cannot possess the machinery required to separate lipid bilayers to allow EV release. This argument applies to all bacteria with the exception of Gram-negative bacteria because these possess an outer membrane layer, whose existence demonstrates that lipids can be exported from the cell and offers a straightforward location for EV release.

Our first argument is that every time a bacterium replicates, it separates mother and daughter cell into isolated cells, *de facto* creating two isolated lipid bilayer compartments, thus demonstrating the presence of membrane fission machinery. Given the fast replication times of the bacteria such a *Staphylococcus aureus* (estimated at 30 min) we assert that lipid bilayers can be separated quickly and efficiently by bacteria. Another instance requiring membrane compartmentalization in bacteria is spore formation. This process requires membrane fission machinery and some of the key players are identified, for example, the protein FisB is required for correct fission in *Bacillus* spp. [19].

Our second argument is that the absence of classical organelles cannot be interpreted to signify bacteria are unable to execute complex membrane dynamics [20–22]. There are instances of organelle-like structures and sophisticated compartmentalization. The fresh-water bacteria *Gemmata obscuriglobus* possesses deep membrane invaginations, a structure reminiscent of a nucleoid [23] and an endocytosis-like process [24]. Magnetosomes, the organelles capable of detecting magnetic fields for spatial orientation in *Magnetospirillum* spp., are invaginations of the cellular membrane [20,21,25]. Intracellular vesicles have been observed by electron cryotomography in several genus of Gram-negative bacteria [13]. In some bacteria membranous structures extend outwards of the bacterial cell body. In other instance, membranous nanotubes are found bridging neighboring cells in Gram-positive [26,27] and Gram-negative bacteria [28,29]. At least one species of *Vibrio* has a membranous sheath in its flagella [28]. This flagella release lipopolysaccharide-containing OMV which gives the appearance of ‘beads-on-a-string’ [29].

Overall, there is abundant evidence of specialized lipid compartments in bacteria while how all these lipid structures arose is still unknown. We conclude that bacteria possess the machinery for complex membrane dynamics which indicates bacteria can possess machinery for EV formation and release.

### Vesicles are artifacts of lipid self-aggregation or debris from lysed cells or waste products

This argument rests on the well-known tendency of lipids to self-aggregate and dismisses EVs as structures that form spontaneously from lipids released from growing and/or dying microbes. If this explanation was correct, then EVs would be more abundant when microbes are heat killed or physically disrupted, since lipids are released from lysed cells. From this, we derive that EV lipid composition should be largely similar to the bacterial cellular membrane and that EV content should represent the composition of extracellular media which happened to become trapped into the self-aggregating lipid bilayers. Firm resolution of this criticism would require direct comparison of cell debris and EVs as well as the determination of the capacity of these released lipids to spontaneously form vesicles.

**Figure 2.**
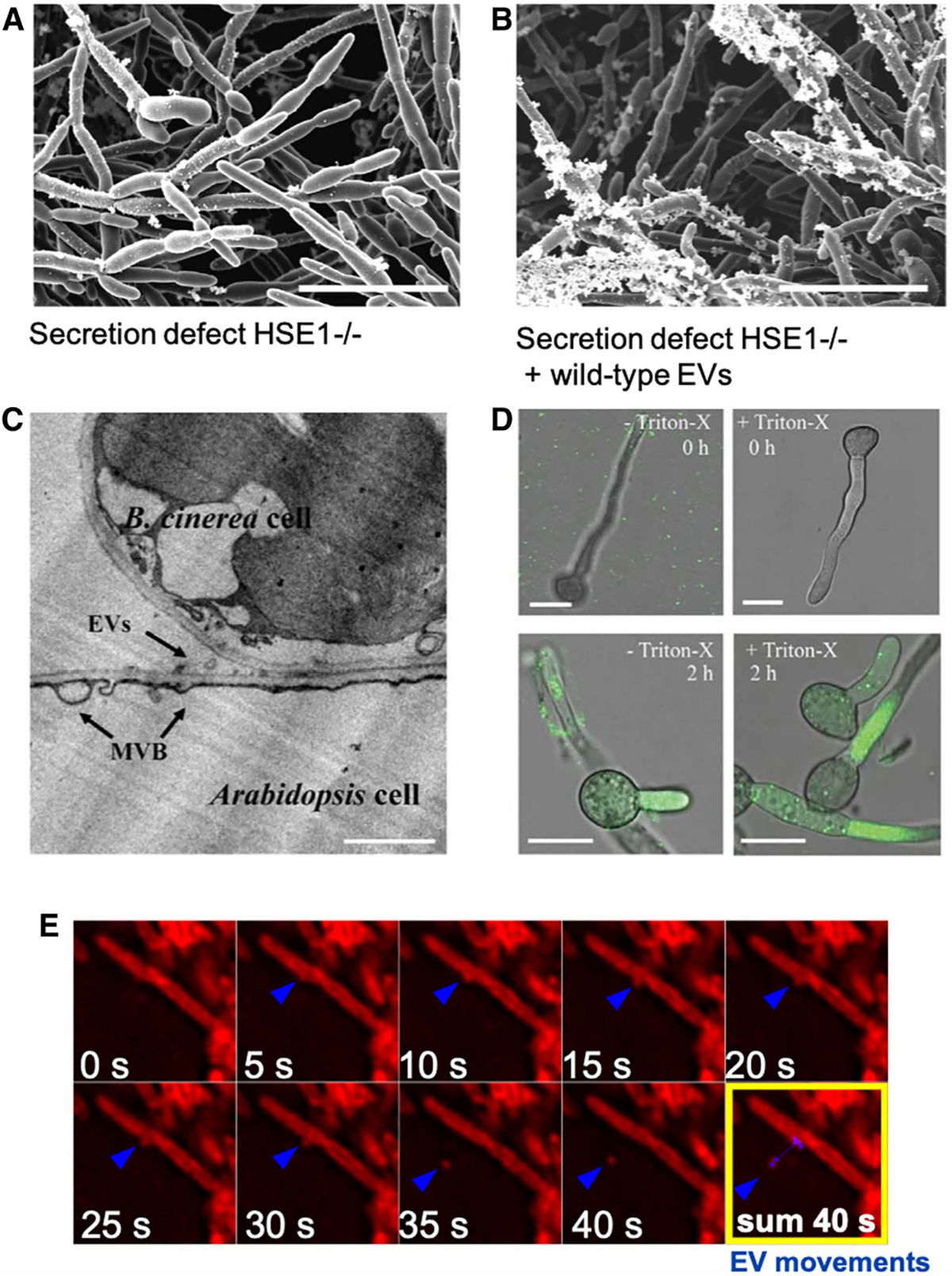
EVs serve as delivery devices in intraspecies and interspecies interactions. (**A**) *C. albicans* deleted for ESCRT subunit HSE1 is defective in biofilm and extracellular matrix. (**B**) Biofilm and extracellular matrix formation were rescued when wild-type EVs were. Added to HSE1−/− strains. Scale bars 11 μm. (**C**) EVs from the plant *Arabidopsis* near the pathogen *B. cinerea* infection sites. Scale bars, 1 μm. (**D**) Isolated Tetraspanin8 –GFP-labeled *Arabidopsis* EVs were taken up by *B*. *cinerea* within 2 h of co-incubation. Scale bars 10 μm. (**E**) *L. monocytogenes* release EVs when infecting a mammalian host. Bacterial phospholipids were previously loaded with fluorescent dye Bodipy-C12 fatty acid which is incorporated and released in EVs. Images courtesy of Zarnowski et al. [11] (**A** and **B**), Cai et al. [47] (**C** and **D**), and Coelho et al. [30] (**E**).

Experimental evidence has largely refuted the view of EVs as artifacts or debris. Performing mock extractions with killed organisms did not isolate EVs [30]. Lipid composition of EVs is different from the lipid bilayer it originates from, observed when comparing EVs with the plasma membrane of whole cells in Gram-positive bacteria *Listeria monocytogenes* or *Streptococcus* [30–32] as well as when comparing EVs with the outer membrane of Gram-negative bacteria [33]. The same is found for RNA cargo, where RNA cargo does not reflect the RNA content of intact cells, which is highly suggestive of selective enrichment [31]. While EVs are enriched in proteins directed for secretion, namely secreted virulence factors (reviewed elsewhere [13]), direct comparison of cell-free and EV-depleted supernatant found that EV composition is only 30% similar to the EV-free supernatant in the fungi *Paracoccidioides brasiliensis* and *Candida albicans* [34,35]. Furthermore, experiments adding an extraneous component (easily detectable for high sensitivity) to actively growing bacteria showed no detection of this extraneous component in EVs [36], arguing against the self-aggregation of lipids entrapping the components of the media. Finally, EVs from wild-type *C. albicans* could reconstitute biofilm formation to strains lacking genes involved in secretion [11] (Figure 2). This study shows EVs are not debris, EVs carry important cargo and deliver that cargo to revert the phenotype caused by certain genetic defects.

Another related argument used by naysayers is ‘EVs are just cellular waste’. To refute this argument, we would plainly state that efficient excretion is critical for a healthy cell and that every organism spends a considerable amount of energy to efficiently sort and process its waste. Therefore, it is critical to understand waste and excretion and it is reasonable that EVs can be part of such a process, and warranting research into EV. Curiously, EV release is increased in conditions of cellular stress, such as exposure to bacteriophages or antibiotics, which perturb cell wall or membranes [37]. In *S. aureus*, EVs formed by phage lysis or antibiotic treatment have different compositions [38]. While it is not clear if this is increased waste to cleanse damaged cellular components or a special case of EVs formed by autolysis (reviewed in [9]), EVs formed in conditions of stress are distinct from EVs from unperturbed cells. Direct comparisons between stress-EVs, lysis-derived EVs and active-growth EVs are still underway.

In summary, multiple studies show composition and cargo of EV are distinct from the microbial cell it originated from and that conditions of stress lead to a different EV cargo. These studies are consistent with EVs as specialized compartments of microbes regulated and adapted to the growth and stress conditions, and thus are not debris or artifacts and possibly a well-organized mechanism to release toxic waste.

### There are no null mutants, which argues against a regulated process

EV detractors have interpreted the difficulty of identifying of EV-null mutants to mean that EVs are an artifact, and a process lacking genetic regulation. We note it is not possible to construct cell-membrane null cells and yet the existence of the cell membrane is accepted, suggesting a logical inconsistency for those who deny EVs due to the absence of known EV-null strains. One explanation for the difficulty in isolating EV-null mutants is that EVs are not a single population, consistent with their observed heterogeneity [4], thus one genetic deletion would abolish only a particular EV subpopulation. Another plausible explanation for the absence of EV-null mutants is that EV formation may share pathways with essential cellular processes like cellular membrane formation. Finally, one could hypothesize that the lack of EV-null strains signifies EVs are essential to the cell, i.e. null mutants do not exist because EVs are vital (see below for roles of EVs in cellular homeostasis). Our view is that there is enough evidence to ponder that EVs are a vital component of microbial cells, while it remains to be revealed what role EVs play such that they are a vital component of microbial cells (see below). This conundrum could be resolved with the engineering of conditional or inducible deletion strains. In this regard, hypersecretor and hyposecretor strains have been found [36,39,40] (to name just a few), and even rare instances of strains with altered EV morphology [41,42], supporting the view that EVs are not an artifact. One last argument: decreased activity of the Yrb ATP-binding cassette transporter system leads to phospholipid accumulation and an increase in OMV in phylogenetically diverse Gram-negative bacteria [39]. These data suggest a conserved mechanism of EV release, at least within Gram-negative bacteria [39]. Therefore, the difficulty in identifying EV-null mutants should not be interpreted as proof of EVs as an artifact, as there are many other possible explanations, as well as a considerable data starting to unravel the molecular machinery of EV release.

### Vesicles cannot cross rigid cell walls

The microbial cell wall protects the cell from mechanical and osmotic stress. Thus, many view the cell wall as a rigid structure and have trouble conceiving how EVs can traverse it. However, it is important to remember that the cell wall remodels itself with every cell division and in response to stress, while simultaneously serving as a conduit for bidirectional traffic of vital supplies. Nutrition requires cell walls to be permeable to a wide range of (macro)molecules. In reality, the cell wall consists of interwoven fibrils and is incredibly elastic. Studies of the yeasts *Schizosaccharomyces pombe* and *C. albicans* showed that the cell wall fibril network can double thickness in a matter of seconds [43,44]. Recently, liposomes containing Amphotericin B and 15 nm gold beads were shown to cross the cell wall of fungi. This uptake was mediated via the ergosterol binding capacity of Amphotericin B [45]. Because this study focused on the intake of liposomes with sizes smaller than EVs, one cannot directly extrapolate to export of EVs, and the mechanism of EV journey through cell walls is still predominantly unknown (reviewed in [46]). However, this study demonstrates that the cargo of lipid droplets significantly influences uptake and cell wall permeability is dependent on the cargo traversing it. Finally, when *Arabidopsis* plants encounter the *Botrytis inereal* fungal pathogen, the plant produced vesicles at the contact site and these EVs are internalized by the fungal pathogen [47] (Figure 2). In this case, an EV like structure and its cargo crossed two cell walls (the plant and the fungi). Overall, we conclude that the mechanisms of EV traversing are unknown. This gap in knowledge cannot be used to state that EVs do not exist and multiple observations support that EVs can cross membranes.

### EVs serve no function and their release is a misuse of energy

An EV release system has multiple advantages over other secretion systems. EVs are posited to function as a primordial excretion mechanism, accumulating and allowing rapid disposal of protein aggregates/unfolded proteins [48] and possibly other toxic compounds. EVs are also implicated in cell wall remodeling in microbes [49] and in plants [50]. Cell wall and EV trafficking are intimately connected since EV release likely requires cell wall remodeling or, at the very least, its elastic distortion. Consistent with this cell wall remodeling function, EVs are frequently observed at the cell division or mother-daughter site [51]. Both of these functions would substantiate why EVs are essential for microbes.

Secretion or excretion of cargo in a concentrated form and protected by a lipid bilayer has significant advantages. Toxic compounds destined for excretion can be isolated within the intracellular milieu to prevent further damage to the microbial cell or to overcome solubility problems. Concomitantly, the contents of EVs are also protected from degradation, for example from proteases or nucleases. Communication via EVs allows delivery of a concentrated but also a more complex message, a key opportunity for cooperation between groups of enzymes. In mammals, osteoblasts release EVs during bone remodeling, which supports the view that EVs are an effective solution to the degradation of complex structures [52], allowing multiple cycles of degradation and rebuilding.

EVs can also function as communication devices in microbial communities [53], such as biofilms [11] or quorum sensing. EVs similarly influence interspecies relationships [54], including predatory and host-pathogen interactions [8,13]. The same advantages of EV-type secretion described above apply to intercellular and interkingdom communication. Precious compounds are protected from degradation, allowing longer life-span and distant dissemination. In pathogenic microbes, EVs often carry a high proportion of virulence factors allowing a high local concentration of the toxin or virulence factor. Whereas secretion of toxins at the cell membrane would result in rapid dilution with diffusion, packaging proteins and virulence factors in EVs allow the delivery of a concentrated punch to the target cell membrane. This cooperation could be analogous of the antigen-MHC cargo in exosomes allowing for a more complex message to be delivered in the immune synapse [55].

Other functions of EVs are still being uncovered. For example, it is unclear whether the increase in EV release in bacteria following phage infection is a defensive decoy against phage infection, or whether EVs are hijacked to potentiate dissemination of phages [9]. It is also intriguing to consider if EVs play a role in nutrient acquisition. In low iron conditions, *M. tuberculosis* increases the release of EVs with a siderophore cargo [56]. This finding suggests that EVs assist in the acquisition of iron from the extracellular milieu, but it remains unclear how the iron–siderophore complex could subsequently be recovered by the bacterial cells. In *Pseudomonas aeruginosa*, the TseF protein facilitates iron delivery from OMV to bacterial cells [57], and finding analogous mechanisms in other microbes [39] would be of tremendous importance. EVs found in seawater support the growth of other microbes, providing the tantalizing suggestion that EVs have a role in carbon flux of marine ecosystems [22]. The role of EVs in nutrient acquisition remains one of the unsolved EV mysteries.

To fundament the criticism of ‘EVs serve no function’ some have argued that EVs cannot travel far enough to perform extracellular functions. In fact, the stability of microbial EVs and how stability impacts EV function are poorly understood. While EVs from *C. neoformans* and *B. anthracis* lyze when exposed to serum albumin [58], EVs from *C. gattii* serve as a relatively long-range communication when interacting with mammalian macrophages [59]. In certain conditions, microbial EVs have remarkably long stability: EVs from gut bacteria were found in the bloodstream of their hosts [16], and EVs can be isolated from seawater [22]. Though these observations may seem contradictory, they may be explained by cargo and composition differences that will have to be clarified in the future.

## Conclusion

The discovery of EVs presents several advantages to secretion since compartmentalization in EVs isolates and protects the contents, as well as expedites simultaneous delivery of different cargos. While there is still much to be discovered in EV biology, the growing body of work in microbial EVs refutes them as mere culture artifacts. If this review leaves you unconvinced, we appeal that you communicate your argument to us. An open debate will inform critical experiments to clarify any conundrums. Finally, we hope this essay helps to advance discussions of the biological processes underlying EVs.

## Perspectives

- EV release in microbes (archaea, bacteria and fungi) is a process involved in myriad steps of microbial life such as cell wall remodeling, cellular division, biofilm formation and intercellular communication. EVs have been identified in all classes of microorganisms and may represent a universal solution to secretion.
- EVs carry proteins, including toxins and virulence factors, as well as nucleic acids and metabolites enclosed by a lipid bilayer and serve as carriers for extracellular remodeling and communication. It is also possible that EVs can serve as an excretion mechanism, i.e. that EVs isolate harmful cellular waste that can be excreted swiftly. EVs from pathogenic microbes have the potential to be harnessed as vaccines and for diagnostics.
- Future directions will unravel (a) mechanisms for EV release as well as how the cargo is targeted to the EVs. This would allow to establish EV-specific markers. (b) How EV release facilitates cooperation and synergy between the cargo. (c) New technologies for isolation, labeling and characterization of EVs.

## Abbreviations

EVs: extracellular vesicles
OMV: outer membrane vesicles
